# Predicting the finished fabric width and areal density (Grams per Square Meter) of commercially produced plain Single Jersey (100% Cotton) Knitted Fabric using Fuzzy Inference System (FIS)

**DOI:** 10.1371/journal.pone.0345720

**Published:** 2026-07-09

**Authors:** Md. Yasin, Abdullah Ibna Rahman, Sheikh Yousuf Abdullah, Shah Alimuzzaman, A. T. M. Faiz Ahmed

**Affiliations:** Department of Fabric Engineering, Bangladesh University of Textiles, Tejgaon, Dhaka, Bangladesh; King Mongkut's University of Technology North Bangkok, THAILAND

## Abstract

The purpose of this research is to predict Finished Fabric Width (FW) & Areal Density (GSM) of 100% cotton plain single jersey knitted fabric by building a fuzzy inference model incorporating key input parameters such as Stitch Length (SL), Yarn Fineness or Count (YC) and Machine Diameter (D). More than 30,000 mass production-grade data points have been used to generate the model with remarkable precision. Once the model was prepared, it was verified using new experimental data. The Coefficient of Determination (R^2^), Mean Absolute Percentage Error (MAPE), and Root Mean Square Error (RMSE) between the actual and the predicted FW were found to be 0.979, 1.214%, 1.103, respectively. For GSM the corresponding metrics were 0.940, 1.661%, 3.892, respectively. Both prediction outcomes showed excellent precision, justifying the model's applicability in the textile industry for predicting two important knit fabric parameters namely FW and GSM. The system's reliability was ensured by using a large set of industry standard data. This, combined with the adaptation of carefully designed fuzzy logic rules based on proven scientific method, significantly contributed to producing more accurate results. Together, all these aspects make the system stand out from similar studies, offering a practical and trustworthy approach for real world textile application with enhanced process optimization.

## Introduction

The production of Knit Fabric has been used in the textile industry for more than 400 years and is one of the most popular methods of fabric production behind weaving [1]. The clever system of intermeshing loops of yarns into a flexible planar substance combined with excellent drapability and breath-ability, altogether makes it the preferable choice of consumers for wearables. As a result, the already blooming industry of knit fabric production has skyrocketed in production capability thus making it more dependent on precision and quality parameters.

In Circular knitting Technology, two of the commonly maintained and often struggled parameters are Finished Fabric Width (FW) and Areal Density (GSM-Grams per Square Meter). While determining the cost of a garment unit, at least 50–60% is consumed by the fabric. As a result, it is a natural requirement to make the best use of it. An efficient marker planning is compulsory to maintain the highest use of fabric. As marker efficiency directly corresponds with fabric width, a predetermined width needs to be maintained in the finished fabric [2]. As for the Areal Density, commonly denoted as GSM (Grams per Square Meter), it directly controls the weight of the fabric. It results in determining the drape, strength, thickness, permeability, weather adaptivity, consumption, costing etc. and more of a fabric. So, a dire need is always there to maintain, control and predict these physical properties.

Parameters like, Fabric Construction, Stitch Length (SL), Machine Gauge (G), Machine Diameter (D), Yarn Count (YC) etc. control the physical, physiological, and mechanical properties of a knit fabric [3,4]. Even changing one of these parameters while keeping others constant can turn two fabrics non-identical. These parameters are controlled and maintained commercially as per buyer and consumer requirements. Two of the most common physical properties that these parameters control are Finished Fabric Width (FW) and Areal Density (Grams per Square Meter-GSM).

Even though knitting technology has been fairly saturated and well-established over the course of many centuries, the control of these parameters to achieve exact properties (FW and GSM) is still dependent on experienced inputs and trial and error processes. In terms of commercial development methods, tolerance range is below 5% in case of both Finished Fabric Width (FW) and Areal Density (GSM).

The full process of knit fabric development consists of obtaining greige fabric directly from knitting machines then generally ends up going through dyeing, finishing, and washing process until the required quality is achieved. Under these process circumstances yarn and fabric end up in a relaxed and natural state thus changing the initial parameters associated with the fabric development [5]. As a result, the theoretical approach to predict physical properties, in this case, Finished Fabric Width (FW) and Areal Density (GSM) has always come short of accuracy. So, knitted fabrics being dimensionally way more unstable and unpredictable when compared to woven fabrics, prone to fall off the tolerance ranges easily.

To solve the issue, Industries tend to store data from previously developed fabrics or follow a long, time-consuming, and costly sample development process with multiple trial and error handling. Most of the time, the data stored is proprietary to specific industry system and sometimes impossible for others to even replicate. Moreover, the repositioning of such data requires a careful data management system with a skilled workforce. This resulted in the growth of demand for such a system that can predict these parameters with pinpoint accuracy. But the outcome of Finished Fabric Width and Areal Density relative to Fabric Construction, Stitch Length (SL), Machine Gauge (G), Machine Diameter (D) and Yarn Count (YC), is irregular, imprecise, and vague.

In recent times different researchers have attempted to use Fuzzy Inference System to predict different physical and mechanical properties of circular knitted fabrics. Haque et al. have different successful attempts at predicting properties like bursting strength and whiteness index using FIS (Fuzzy Inference System) [6,7]. While determining the Bursting Strength of cotton plain knitted Fabrics, Ertuğrul and Uçar have used Multilayer Perceptron (MLP) combined with ANFIS (Adaptive Neuro-Fuzzy Inference System) and obtained satisfactory results [8]. Vadood M. has also used ANFIS (Adaptive Neuro-Fuzzy Inference System) to predict the color index quality of acrylic fiber and has claimed to obtain better prediction results while compared with linear regression modelling system [9]. Another attempt by Akter Smrity et al. at predicting the Dimensional Stability (Length-Width wise Shrinkage Tendency) and Areal Density (GSM), has successfully utilized the FIS and achieved good results [10]. Haroglu D. has developed Artificial Neural Network (ANN) and Fuzzy logic (FL) model for predicting the air permeability of pile loop fabrics, produced by using textured polyethylene terephthalate (PET) yarns from different filament fineness [11]. Besides knit fabrics the fuzzy logic has also been used for woven fabrics to predict different qualities. For example, Khalil E and Akter M have applied fuzzy logic effectively to predict seam strength in cotton plain canvas fabrics, using thread linear density and stitch per density as key inputs. The Mamdani fuzzy inference system achieved high accuracy, with R^2^ values above 0.98 and MAPE below 5%, demonstrating robustness in modeling warp and weft seam strength. This study highlighted the growing role of fuzzy systems in enhancing quality control and supporting automation in textile manufacturing [12]. Maruf Hasan S et al. have also developed Fuzzy Logic Expert System (FLES) to model the effect of thread density on the thermal properties of plain-woven cotton fabrics. Using PPI and EPI as inputs, the system accurately predicted CLO, thermal conductivity, and thermal transmittance, achieving R^2^ values above 0.95. This study highlighted FLES as reliable alternative to ANN and ANFIS, requiring fewer data while maintaining strong predictive accuracy [13]. While analyzing the performance of natural dyes on Jute-cotton blended fabrics Hassan M et al. have used fuzzy logic for quality evaluation [14]. Kodaloğlu F et al. developed four fuzzy models based on fabric fiber blend ratios and knitting structures, providing insights into energy-efficient drying. This approach has highlighted the potential of fuzzy systems in reducing cost and energy use without compromising fabric quality [15].

So, it can be said with certainty that the use of FIS has seen its successful phases at the textile domain. However, most of the studies have used data generated under Lab based or controlled environment. Such conditions may make the results difficult to be replicated under industry standards, especially because of the difference in scale and quantity. This also limits the data to be referenced with real world practical data as in most cases only a few datasets are used to back each dependency. This in return might reduce the accreditation of the results.

The Fuzzy Inference System may exceed at extracting results with minimal data availability but that is also why it requires highly accurate datasets. Most of the studies have relied on minimal number of fuzzy regions, generating only a handful of rules, thus raising questions on model coverage and compactness. This is certainly because of the insufficiency of data and unscalable nature of lab grown datasets. Lab documented or controlled datasets are often disregarded in practical environment, as they fail to match with the results of mass production-scale data and show discrepancies [16,17]. That is especially because of the production scale and quantity that differs from lab-scale. Bangladesh, being a country vastly industrialized by textile realm, has the abundance of industry standard datasets that are based on past trial and errors and are used to manually determine, calculate, and predict different Fabric Properties, in this case Finished Fabric Width (FW) and Areal Density (GSM).

So, in a condition where data collection is uncomplicated, a Modified version of Fuzzy Inference System that uses the abundance of data, accompanied with the 5 step Fuzzy Rule Generating process from Examples, propositioned by Wang and Mendel can be combined to produce exceptionally good result [18]. The plan is to break down crisp datasets into small clusters, finding the datasets that are most prominent for that specific cluster and then generating rules using the Wang and Mendel method. This theoretically will ensure that each rule is generated following scientific method with very good numbers of practical datasets to back its accuracy.

Fuzzy logic is a modification in traditional Binary, Boolean or Crisp Logic system that was first proposed by Lutfi A. Zadeh [19]. Fuzzy Logic works with approximation, unlike the traditional system that deals with objects by exact values like One (1) or Zero (0), True or False, Yes or No, On or Off. It uses Fuzzy Sets to part the objects into Membership Functions (MFs) and assign continuous degree of value ranging from 0 to 1 [20]. In a sentence, Fuzzy Logic introduces the shades of grey between black and white [21].

**Fig 1:**
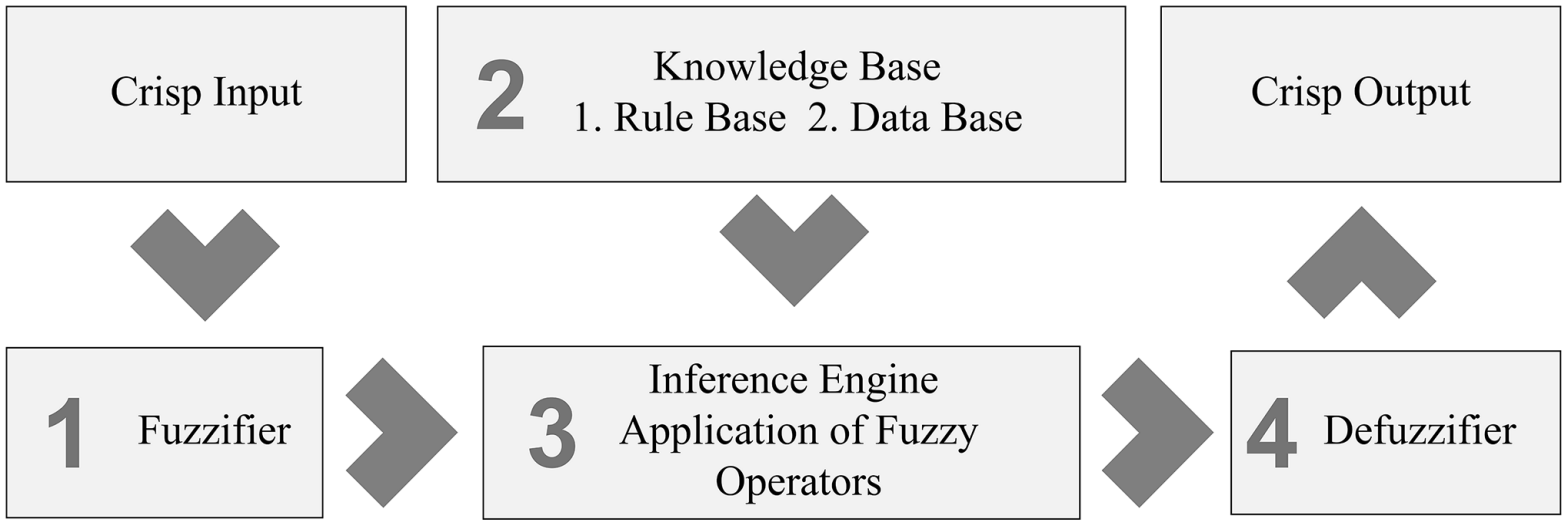
Primary Modules of Fuzzy Inference System.

The Fuzzy Logic control system contains four primary modules [22] as shown in **Fig 1**. The Fuzzifier module takes the crisp values of an experiment and fuzzifies the values. The process is done by taking the crisp values as an input and determining how strongly it fits into the fuzzy sets using the Membership Functions. Even though multiple types of membership functions are available, e.g., Sigmoid, Trapezoidal, Gaussian, Bell Shaped etc. the most used ones are the Linear Membership Functions (Linear-S, Triangular and Linear-Z) for their simplicity, computational efficiency and robustness to noise and input variations [23].

The Triangular Membership Functions are defined as,


$$\begin{array}{c} f_{T}(x)\;=\;\left\{ \begin{array}{c} @c\;\;\;\;\text{0};\;if\;x\leq p\\ \frac{x-p}{q-p};\;if\;p\leq x\leq q\\ \frac{r-x}{r-q};\;if\;q\leq x\leq r\\ \;\;\;\;\;\;\;\text{0};\;if\;x\geq r \end{array}\right.\; \end{array}$$ 1

Equation 1 shows the calculation for determining the fuzzified value for a triangular membership function. For any crisp input x, $f_{T}(x)$ is the fuzzified value and p, q, r are the starting, peak, and terminating values of the Membership Function respectively.

The Linear S-shaped Membership Functions are defined as,


$$\begin{array}{c} f_{S}(x)=\;\left\{ \begin{array}{c} @c\;\;\;\text{1};\;if\;x\leq p\\ \frac{q-x}{q-p};\;if\;p\leq x\leq q\\ \;\;\;\;\;\;\;\;\text{0};\;if\;x\geq q \end{array}\right.\; \end{array}$$ 2

The Linear Z-shaped Membership Functions are defined as,


$$\begin{array}{c} f_{Z}(x)=\;\left\{ \begin{array}{c} @c\;\;\;\text{0};\;if\;x\leq p\\ \frac{x-p}{q-p};\;if\;p\leq x\leq q\\ \;\;\;\;\;\;\;\;\text{1};\;if\;x\geq q \end{array}\right.\; \end{array}$$ 3

Equation 2 and 3 respectively shows fuzzification process of Linear S-shaped and Linear Z-shaped membership function, where the functions are specified between p and q. $f_{S}(x)$ and $f_{Z}(x)$ are the fuzzy values for any crisp input x.

The Knowledge Base that comprises Data Base and Rule Base connects the Input with the Output of the model [24]. That is why it's called the Heart of the model. As such, accurate and practical rule formulation is important. In fuzzy logic module, two of the most effective and efficient systems are Mamdani and Sugeno fuzzy inference system. These two inference systems differ in Rule Base, in Output Representation, in Defuzzification etc.

**Mamdani rule:**


$$\begin{array}{c} \text{If a is P and b is Q},\text{ then c is R} \end{array}$$ 4

**Sugeno rule:**


$$\begin{array}{c} \text{If a is P and b is Q},\text{ then c is f}(\text{x},\text{ y}) \end{array}$$ 5

In equation 4 and 5, a and b are the input variables and, c is the output variable with P, Q and R being the fuzzy sets in case of Mamdani rule system. The output of Sugeno rule system is a first-degree polynomial function, f(x, y), which is a crisp output value. The preceding portion (IF part) of the rule sets the condition, and the following portion (THEN part) sets the outcome of the rule.

The Fuzzy rule base creates a controlled structure which is combined using the knowledge of individual operator. So, a system that is easy to understand, easy to modify and reasonable enough is preferable to develop a complete rule base. That is where the Mamdani fuzzy inference system shines as it is both simple to comprehend and easy to understand [25–27]. While using Mamdani Inference system the output is a Fuzzy Value that needs to be defuzzified into crisp values, compared to Sugeno Inference System that always outputs a crisp value using the Weighted Average method. The Centroid Defuzzification method, which is mostly used for defuzzifying the Mamdani Model output, reveals the balance point of the total fuzzy set. Comparing the total fuzzy output with a flat plate, its balance point will be the point along x axis, upon which the plate would balance itself. The equation is defined as,


$$\begin{array}{c} c^{*}=\;\frac{\int c.f_{X}(c)dc}{\int f_{X}(c)dc} \end{array}$$ 6

In equation 6, the output c^*^ is the crisp value defuzzified through the centroid defuzzification method. f_X_(c) is the total output fuzzy membership function and c is the output variable.

The rule base of the FIS model being the heart of the system, needs to be well optimized and a systematic approach can be taken to generate Fuzzy Rules using Wang and Mendel’s 5 steps [18]. The first and second step is usual Fuzzification, and Rule generation as shown in Fig 1.

**Step-1**: Dividing the Input and Output Spaces into Fuzzy Regions [18].

**Step-2**: Generating Fuzzy Rules from Given Data Pairs [18].

**Step-3**: Assigning a Degree to Each Rule [18].

However, a large dataset might end up creating conflicting rules and outputs. Wang and Mendel’s proposition is to assign a degree to each rule and selecting the rule with the highest resemblance [18]. For any rule x expressed as, “IF a is P and b is Q, THEN c is R”,


$$\begin{array}{c} D(x)=\;d_{P}(a)d_{Q}(b)d_{R}(c) \end{array}$$ 7

Here, in equation 7, D(x) is the product of the degree of membership functions P, Q and R (denoted as d_P_, d_Q_ and d_R_) for inputs a, b and output c respectively. Each degree of membership function will be a value ranging between 0–1 and will indicate how much an input belongs to the MF. The outcome of this equation helps compare between conflicting rules. This step will enable the formulation of rules to be more accurate and scientific. Steps through 4 and 5 are regular Fuzzy Rule base combination and Defuzzification of the system for crisp output.

**Step-4**: Creating a Combined Fuzzy Rule Base [18].

**Step-5**: Determining the Mapping Based on the Combined Fuzzy Rule Base [18].

This 5-step method helps generate the rules systematically while enabling the choice of most effective and accurate rules. Once the rules are set out, the Inference System takes control. Following the processing through Inference System, the fuzzy output is generated and later defuzzified to crisp human readable values.

The principal purpose of this study can be allotted into three categories, 1) To build a system that can predict the Finished Fabric Width (FW) and Areal Density (GSM) by taking Stitch Length (SL), Yarn Count (YC) and Machine Diameter (D) as inputs; 2) To ensure the accreditation of the system by using a large amount of Industry-Standard dataset; and 3) To formulate the Fuzzy Logic Rules using a proven scientific methodology to generate better output results. The combination of these three features has potentially made the system unique compared to other similar studies. While other studies have found good results using Fuzzy Inference System, they often proposed models with small amount of rule-base, depending on lab or small-scale datasets, and mentioning no method while generating rules, making their outcomes less acceptable towards industry standards. A textile engineer can use our system to potentially replace their whole historical database reposition and predict the quality of finished fabric before starting the process.

## Materials and methods

### Fabrics and yarns

More than 30,000 industry standard practical data of Single jersey plain weft knitted fabrics (from a reputed textile industry in Bangladesh) were used for the experiment. The fabrication process employed 100% cotton yarn, featuring counts ranging from 22 Ne to 34 Ne (English yarn count system).

### Knitting and finishing

24-gauge circular weft knitting machines were used for production where diameter of the machine ranged from 28 inches to 40 inches. Stitch length for each loop (all knit loop) was maintained ranging from 2.65 mm to 3.05 mm. To avoid double digit values after the decimal point, SL was measured for 10 loops changing the range from 26.5 mm to 30.5 mm. This SL measuring system was followed throughout the whole model. 24-gauge machine is most widely used in the industry for its versatility and common application*.* This is because 24G can cover a wide range of Yarn Count and GSM, provide optimal shrinkage result, and increase the spirality and stability performance of the fabric [28,29].

Winch dyeing machines were used for the dyeing, and standard finishing process was maintained where each fabric went through Dryer Machines. The fabrics were Tumble Dried for shrinkage testing and shrinkage was found within ± 5% range in both Length and Width wise direction, which falls within the tolerance limit according to industry standards.

### Finished fabric width and areal density (GSM)

Following the ISO-22198:2006 and ASTM D3776/D3776M-09a(2017) method, the width and GSM (grams/square-meter) of the fabric were determined, respectively. Where the measured Finished Fabric Width (FW) varied between 56–84 inches and GSM ranged from 130 to 181.

### Development of Fuzzy prediction model

The model was developed using the pre-built Fuzzy Logic Toolbox^TM^ add-on of MATLAB^®^ R2023b software developed by MathWorks^®^. For the developments of the project, knitting parameters such as Stitch Length (SL), Yarn Count (YC), and Machine Diameter (D) were used as input variables and Finished Fabric Width (FW), and Areal Density (GSM) were used as output variables. Mamdani Type-1 Fuzzy inference system was used for the system.

#### 2.4.1. Dataset preparation and pre-processing.

For development of the Fuzzy Inference System model, the industry-provided master dataset consisted of more than 35,000 production observations collected from commercially manufactured plain single jersey knitted fabrics. Since the master dataset included both standard industrial production records and some experimental or non-standard parameter combinations, an initial screening process was conducted using purposive (expert) sampling under the supervision of industry specialists. This process removed impractical or non-representative data entries and resulted in a filtered dataset of approximately 32,000 observations. This filtered dataset was considered the raw dataset for model development [30].

#### 2.4.2. Data randomization and train-test split.

Prior to model development, the filtered raw dataset was randomly shuffled using Python’s built-in random library to reduce any ordering bias in the data arrangement. After randomization, the dataset was divided into training and testing subsets. Approximately 24,000 samples (≈75%) were used for development of the fuzzy inference model and rule generation, while approximately 7,500 samples (≈25%) were reserved as an independent test set for model evaluation. The test data were not used during rule generation or model construction.

#### 2.4.3. Step – 01: Dividing the Input and Output Spaces into Fuzzy Regions [18].

**Fig 2:**
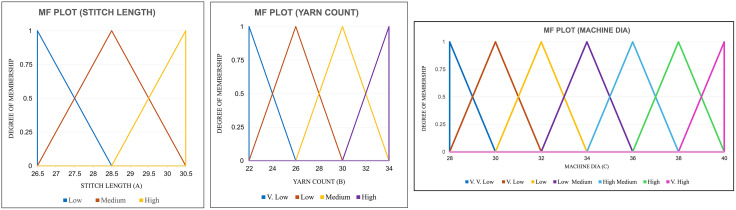
Membership Function Plot of Input Variables, (A) SL, (B) YC and (C) D.

**Fig 3:**
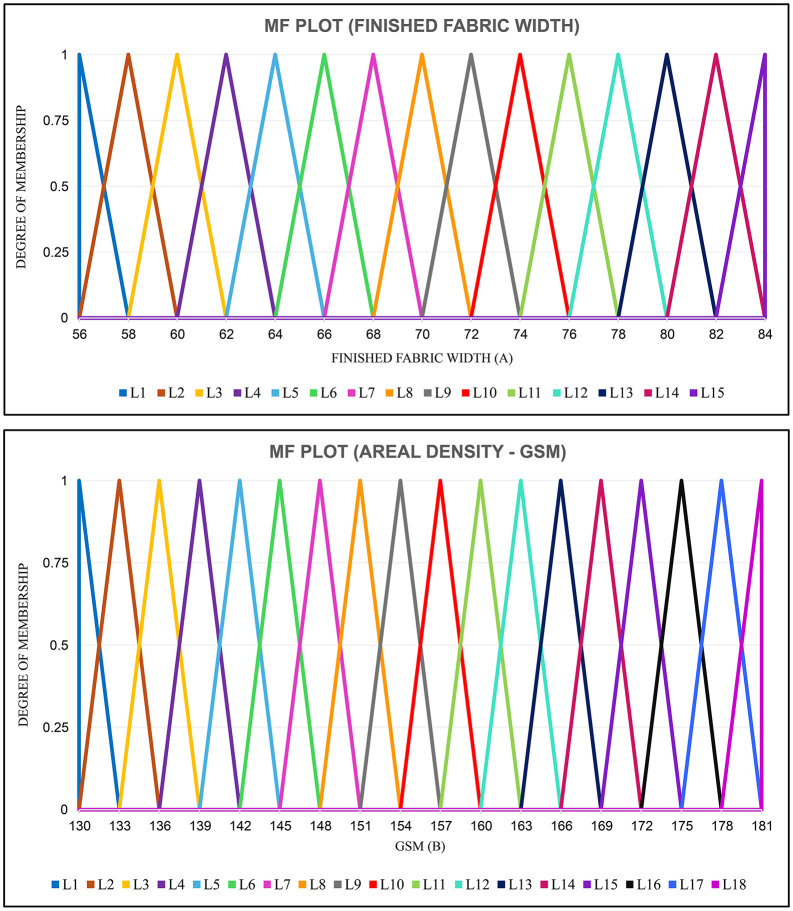
Membership Function Plot of Output Variables (A) FW and (B) GSM.

The input variables, namely SL (Stitch Length), YC (Yarn Count) and D (Machine Diameter) were divided into 3, 4 and 7 MFs (Membership Functions). The full details are mentioned in **Table 1. Membership Functions for Input-Output Parameters**. The starting and terminating values of the MFs were chosen in such a way that they cover the highest extent of the database. The MFs used for the fuzzy prediction models followed three different shapes, where the emerging and ending MFs were Linear S-shaped and Linear Z-shaped respectively and central MFs were Triangular-shaped. These Linear MFs (Linear-S, Triangular and Linear-Z) were chosen for their simplicity yet better performance [23]. These shape-based MFs for the input variables SL, YC, D and output variables FW, GSM were developed using the MATLAB Fuzzy Logic Designer, and are shown in Figs 2(A-C) and in Figs 3(A-B) respectively. And the architecture of the fuzzy inference model is presented in S1 Fig of the Supporting Information.

**Table 1:** Membership Functions for Input-Output Parameters.

Parameters		Range	Membership functions	Type
**Input**	Stitch length (SL) (for 10 loops)	26.5-30.5 (mm)	Low	Linear-S
			Medium	Triangular
			High	Linear-Z
	Yarn Count (YC)	22-34 (Ne)	V. Low	Linear-S
			Low, Medium	Triangular
			High	Linear-Z
	Machine Dia (D)	28-40 (inches)	V. V. Low	Linear-S
			V. low, Low, Low Medium, High Medium, High	Triangular
			V. High	Linear-Z
**Output**	Finished Fabric Width (FW)	56-84 (inches)	L1	Linear-S
			L2, L3, L4, L5, L6, L7, L8, L9, L10, L11, L12, L13, L14	Triangular
			L15	Linear-Z
	Areal density (GSM)	130-181 (GSM)	L1	Linear-S
			L2, L3, L4, L5, L6, L7, L8, L9, L10, L11, L12, L13, L14, L15, L16, L17	Triangular
			L18	Linear-Z

#### 2.4.4. Step – 02: Generating Fuzzy Rules from Given Data Pairs [18].

Although three (3), four (4), and seven (7) membership functions were assigned to the input variables of SL, YC, and D, respectively, leading to a total of 84 possible rules, only 64 rules were generated for the input and output parameters based on collected factory data. The disregarded rules were chosen based on data unavailability. The incompatible rules lacked acceptable datasets in the database to generate their output. This is because the number of possible rules is a numerical value generated through mathematical combination and some combinations are not practically feasible as there are deep relationships between Machine Gauge, Stitch Length and Yarn Count. It's not possible to combine random values of these parameters freely. As a result, any value of SL or YC cannot be used for a certain Machine Gauge [31].

The properties of the whole model are shown in **Table 2**. A Mamdani max-min inference approach was used to organize the Membership Functions into a single fuzzy set.

**Table 2:** Properties of the Model.

**Inputs (3)**	1. Stitch Length (SL)	3 Fuzzy Sets
	2. Yarn Count (YC)	4 Fuzzy Sets
	3. Machine Dia (D)	7 Fuzzy Sets
**Outputs (2)**	1. Finished Fabric Width (FW)	15 Fuzzy Sets
	2. Areal Density (GSM)	18 Fuzzy Sets
**No. of Possible Rules**	84	
**No. of Sustained Rules**	64	
**Type**	Mamdani Type – 1	
**AND Method**	Min	
**OR Method**	Max	
**Defuzzification Method**	Centroid	
**Implication Method**	Min	
**Aggregation Method**	Max	

#### 2.4.5. Step – 03: Assigning a Degree to Each Rule to Resolve Conflicting Outputs [18].

Once the data evaluation and range determination were done, the fuzzy rules were generated and compared with the data sets by allocating degree of membership function to them accordingly. For example, a data set was defined as {SL, YC, D} = {28, 30, 28} and from consecutive Figs 2(A), 2(B) and 2(C), and using equation 1, 2 and 3, SL has degree 0.75 in Medium, YC has degree 1 in Medium, and D has degree 1 in V. V. Low. So, the allocated rule for the data set {SL, YC, D} = {28, 30, 28} would be {Medium, Medium, V. V. Low}. Upon precise filtering of the datasets, maintaining the central tendencies of medium range for SL and YC, and V. V. Low range for D, more than 500 datasets with the output result indicating L1 for FW and L5 for GSM were found. As a result, the rule was generated as,

“IF SL is Medium, and YC is Medium, and D is V. V. Low, THEN FW is L1, and GSM is L5”

Due to the availability of large number of datasets, every rule was backed with numerous examples claiming their accuracy to higher extent.

For evaluating conflicting datasets (having same IF part with different THEN part) e.g., Data Set 1 (DS_1_), {SL, YC, D} → {FW} = {28, 34, 36} → {74} and Data Set 2 (DS_2_), {SL, YC, D} → {FW} = {27.5, 32, 36} → {72} falls in the identical rule base {Medium, High, H. Medium}. But their output levels were different. The 1st set indicating FW at L10, and 2nd set indicating at L9. To solve the issue, using equation 7, and Fig 2(A) for SL, Fig 2(B) for YC, Fig 2(C) for D and Fig 3(A) for FW we get,


$$\begin{array}{c} \text{D }\left({\text{DS}}_{\text{1}}\right)=\;{\text{d}}_{\text{SL}}(\text{28}){\text{d}}_{\text{YC}}(\text{34}){\text{d}}_{\text{D}}(\text{36}){\text{d}}_{\text{FW}}(\text{74}) \end{array}$$

$$\begin{array}{c} \;=\;\text{0}.\text{75 }\times \;\text{1}\;\times \;\text{1}\;\times \;\text{1}\;=\;\text{0}.\text{75 } \end{array}$$

$$\begin{array}{c} \text{D }\left({\text{DS}}_{\text{2}}\right)={\text{d}}_{\text{SL}}(\text{27}.\text{5}){\text{d}}_{\text{YC}}(\text{32}){\text{d}}_{\text{D}}(\text{36}){\text{d}}_{\text{FW}}(\text{72}) \end{array}$$

$$\begin{array}{c} \;=\;\text{0}.\text{5}\;\times \;\text{0}.\text{5}\;\times \;\text{1}\;\times \;\text{1}\;=\;\text{0}.\text{25} \end{array}$$

From the calculation it is evident that degree of DS1 > DS2. So, the choice of data set for rule {Medium, High, H. Medium} would be DS1. Similar method was applied in assigning the rules for GSM, where output levels tend to be conflicting while maintaining the same input MFs. The complete set of generated fuzzy rules is available in S2 File of the Supporting Information.

#### 2.4.6. Step – 04: Creating a Combined Fuzzy Rule Base [18].

The formation of a combined fuzzy rule base was done in the Fuzzy Logic Designer app rather than conventional box method.

#### 2.4.7. Step – 05: Determining the Mapping Based on the Combined Fuzzy Rule Base [18].

As the Mamdani Fuzzy Inference System provides outputs in fuzzy regions, A defuzzification method, specifically the center of gravity or centroid defuzzification method was applied to transform the fuzzy output into a non-fuzzy crisp numeric value. Use of equation 6 converted the fuzzy output into crisp human readable values.

**Fig 4:**
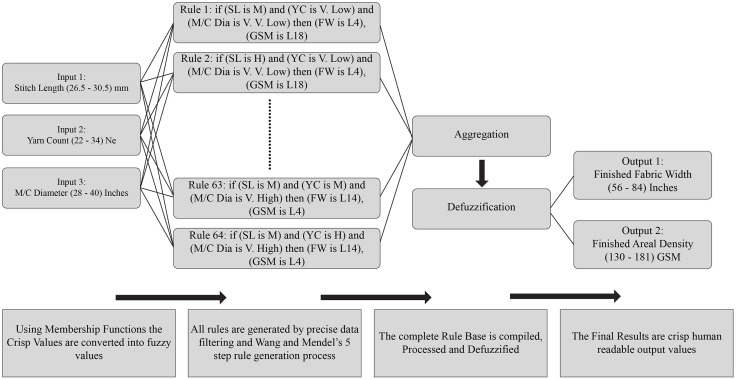
Schematic diagram of fuzzy modeling for predicting FW and GSM.

The schematic diagram of the Model is shown in **Fig 4**.

### Prediction Metrics

The proposed model was investigated according to accredited standards such as Mean Absolute Percentage Error (MAPE), Root Mean Square Error (RMSE), and Coefficient of Determination (𝑅^2^) for determining the prediction performance of the model. The formulas of accuracy measurements were given in equations 8, 9 and 10 respectively.

MAPE helps determining the difference between actual and predicted value as a percentage of error through regression analysis and the lower the value of MAPE the more accuracy the model offers [32,33]. It is calculated as –


$$\begin{array}{c} \text{MAPE}=\frac{\text{1}}{\text{N}}\;\sum_{\text{i}=\text{1}\;}^{\text{i}=\text{N}}\left(\frac{\left|{\text{A}}_{\text{d}}-{\text{P}}_{\text{d}}\right|}{{\text{A}}_{\text{d}}}\times \text{100}\right) \end{array}$$ 8

RMSE measures the error of a prediction by comparing the actual with the predicted value through determining the squared average of the model errors. The lower the value of RMSE (close to zero) the better the result of the model [34,35]. It is calculated as –


$$\begin{array}{c} \text{RMSE}=\;\sqrt{\frac{\sum_{\text{i}=\text{1}}^{\text{i}=\text{N}}{\left({\text{A}}_{\text{d}}-{\text{P}}_{\text{d}}\right)}^{\text{2}}}{\text{N}}} \end{array}$$ 9

Coefficient of Determination (R^2^) explains how much of the variation in the predicted values can be explained by the actual values. The closer the value to 1 means the higher the prediction accuracy [36]. It is calculated as –


$$\begin{array}{c} {\text{R}}^{\text{2}}=\text{1}-\left(\frac{\sum_{\text{i}=\text{1}}^{\text{i}=\text{N}}{\left({\text{A}}_{\text{d}}-{\text{P}}_{\text{d}}\right)}^{\text{2}}}{\sum_{\text{i}=\text{1}}^{\text{i}=\text{N}}{\left({\text{A}}_{\text{d}}-{\text{M}}_{\text{d}}\right)}^{\text{2}}}\right) \end{array}$$ 10

In equation 8, 9 and 10, A_d_ is the actual value, P_d_ is the predicted value, M_d_ is the Mean value, and N is the number of observations.

## Results and discussion

### Executing the Fuzzy prediction model

**Fig 5:**
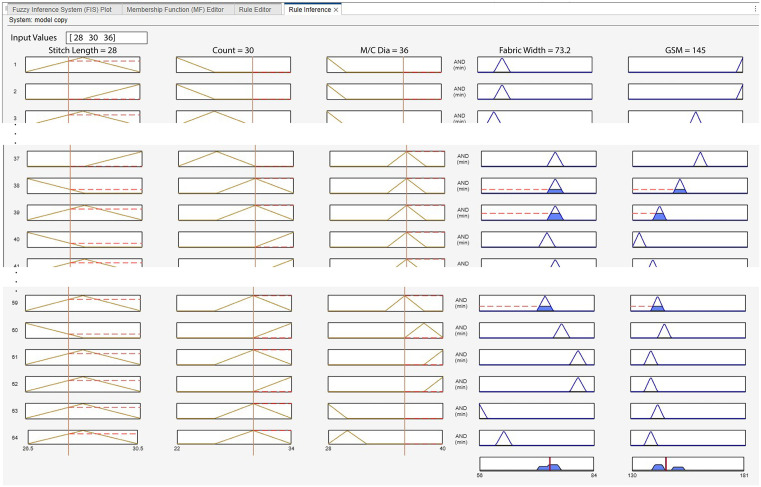
The Fuzzy Inference Model in action.

**Fig 5** illustrates an instance of how the fuzzy prediction model operates. Only one example has been displayed in Fig 5 for ease of discussion. According to the rule, when Stitch Length (SL) is Medium, and Yarn Count (YC) is Medium, and Machine Diameter (D) is High Medium then the predicted Finished Fabric Width (FW) would be at Level 9–10 (L9-L10) and Areal Density (GSM) would be at Level 5–6 (L5-L6).

For example, if Stitch length (SL) was 28 mm, Yarn Count (YC) was 30 Ne and Machine Dia (D) was 36 inches then by reviewing all 64 rules simultaneously with aggregating and defuzzifying, the ultimate Fuzzy model crisp output Finished Fabric Width (FW) was 73.2 inch and Fabric Areal Density (GSM) was 145. Additional model outputs for selected combinations of stitch length, yarn count, and diameter are provided in S2–S7 Figs of the Supporting Information.

### Influence of Input variables on Finished Fabric Width and GSM

#### Influence of Stitch length (SL).

**Fig 6:**
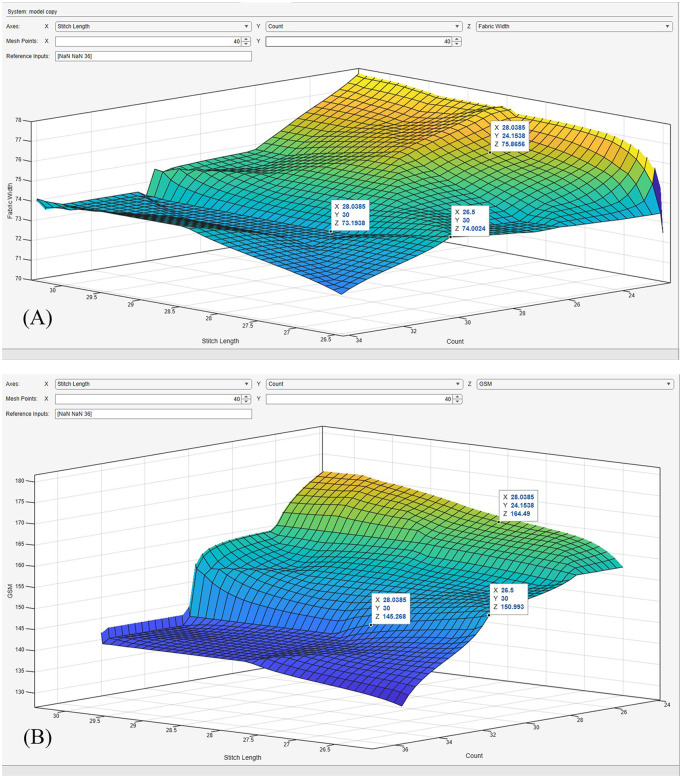
Surface Plot showing the impact of (A) SL and YC on FW, (B) SL and YC on GSM.

The trend in the changing of Finished Fabric Width (FW) and Areal Density (GSM) with the Stitch length could be clearly seen from surface plot (Figs 6A, 6(B) and Fig 8A and 8B,). This effect can also be observed by keeping the other two input variables, Yarn Count (YC) and Machine Diameter (D) constant (in Figs 9A, 9B). The Finished Fabric Width (FW) and Areal Density (GSM) decreased with the increasing value of SL. The result showed that Finished Fabric Width decreased by about 1.21% (which was not significant enough) and Areal Density (GSM) decreased by about 6% when the Stitch length increased from 26.5 mm to 28.5 mm for 30 Ne Yarn Count and 36-inch Machine Diameter (Figs 9A, 9B).

**Fig 7:**
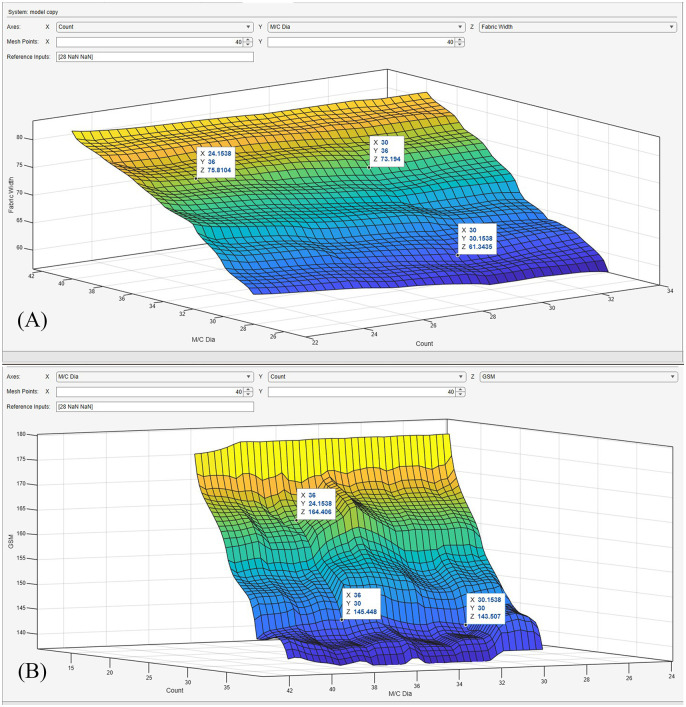
Surface Plot showing the impact of (A) D and YC on FW, (B) D and YC on GSM.

**Fig 8:**
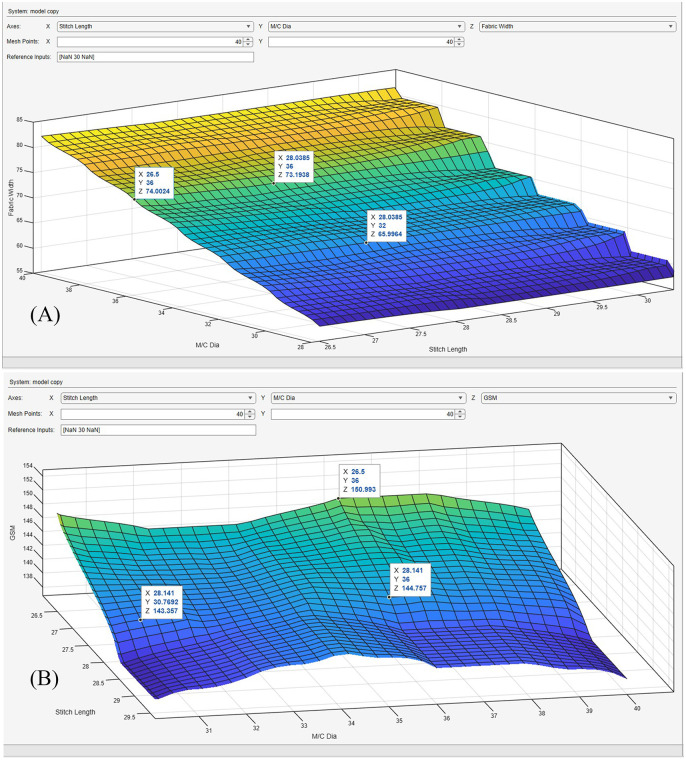
Surface Plot showing the impact of (A) D and SL on FW, (B) SL and D on GSM.

#### Influence of Yarn Count (YC).

**Fig 9:**
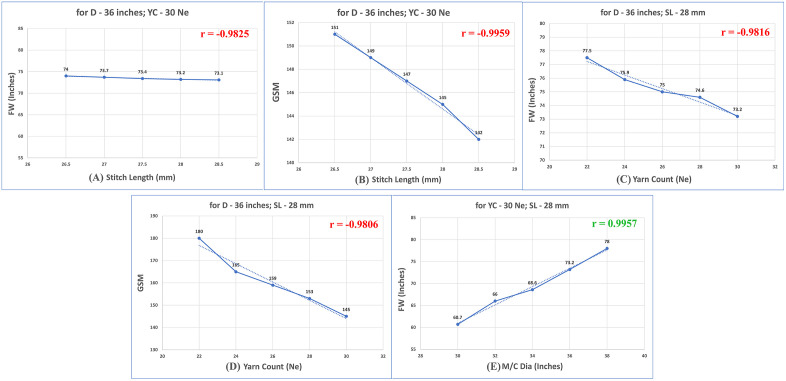
Effect of (A) SL on FW, (B) SL on GSM, (C) YC on FW, (D) YC on GSM and (E) D on FW.

From the surface plot (Figs 6(A), 6(B) and Figs 7(A),7(B)) and the scatter diagram Fig 9C and 9D, the trend in changing of Finished Fabric Width (FW) and Areal Density (GSM) with the Yarn Count could be clearly observed. This effect can be observed by keeping the input variables Stitch Length (SL) and Machine Diameter (D) constant (In Figs 9(C), 9(D)).

As the Yarn Count increased, Finished Fabric Width and Areal Density (GSM) decreased and vice versa. The result showed that Finished Fabric Width decreased by about 5.5% and Areal Density (GSM) decreased by about 19.5% when Yarn Count increases from 22 Ne to 30 Ne for 28 mm Stitch length and 36-inch Machine Diameter. (Figs 9(C), (D)).

#### Influence of Machine Dia (D).

From the Surface plot (Figs 7(A), 7(B) and Figs 8(A), 8(B)) and the scatter diagram (Fig 9E), a clear trend was observed in the variation of Finished Fabric Width (FW) and Areal Density (GSM) with Machine Diameter (D), where a raise in machine diameter results in a corresponding increment of Finished Fabric Width (FW). This effect can be observed by holding the input variables, Stitch Length (SL) and Yarn Count (YC) constant (Fig 9(E)). The result showed the Finished Fabric Width increased by about 28.5% when Machine Diameter increased from 30 inches to 38 inches for 28 mm Stitch length and 30 Ne Yarn Count (Fig 9(E)).

It was also visible that by keeping other parameters constant and only changing the Machine Diameter (D), it didn't contribute significantly to the change of Fabric Areal Density (GSM) (Figs 7(B), 8(B)). This is because GSM of the fabric primarily depends on input factors such as Yarn Count, Stitch Length and Machine Gauge.

To mechanistically understand why machine diameter influences the overall width of the knitted fabric but does not affect the fabric weight per unit area (GSM), it is necessary to examine the dimensional parameters of the structural knit-cell (SKC). The SKC represents the smallest repeating unit of any knit structure [37].

The overall width of a fully relaxed knitted fabric is determined by the total number of needles in the machine (N), the structural cell stitch length (SCSL), and specific dimensional constants (n_w_: The number of needles forming the width of the structural knit-cell, and K_3_ = Wales per unit cm × SCSL) [37]. This relationship is defined mathematically as shown in equation 11 [37]:


$$\begin{array}{c} \text{Fabric Width}=\frac{N(SCSL)}{n_{w}K_{\text{3}}} \end{array}$$ 11

The variables in this geometric model dictate the physical bounds of the fabric. Because the total number of needles N is inherently linked to the machine's diameter for a specific gauge, an increase in machine diameter directly scales the value of N, thereby increasing the overall fabric width but by no means affects the fabric weight.

Conversely, the areal density of the fabric (GSM, or weight in grams per square meter) is determined by the mass of the yarn contained within the repeating knit-cells [37]. The formula as shown in equation 12 for fabric weight per square meter is given by [37]:


$$\begin{array}{c} \text{Weight of Fabric}/m^{\text{2}}=\frac{\text{0}.\text{1}K_{\text{1}}tex}{SCSL} \end{array}$$ 12

Where, tex: The linear density of the yarn, and K_1_: The dimensional constant for the fully relaxed state of the specific fabric structure (K_1_ = Courses per unit cm × Wales per unit cm × (SCSL)^2^) [37].

The variables defining areal density are independent of the machine's circumference. This geometric analysis mechanistically proves that while machine diameter controls Number of Needle (N), which is a primary variable in calculating fabric width, it is completely absent from the calculation for fabric weight per square meter. The GSM is exclusively a function of yarn count tex, stitch length (SCSL), and the structural constant K_1_, demonstrating that alterations to machine diameter will not impact the final GSM of the fabric, provided the structural knit-cell parameters remain constant.

The results obtained from the prediction model were well aligned with the practical datasets, where the GSM increased with the decreasing influence of Stitch length and Yarn Count and vice versa. Finished Fabric width significantly increased with the increasing machine dia. It was also found that Machine Dia (D) and Stitch Length (SL) did not contribute to a mentionable change in Areal Density (GSM) and Finished Fabric Width (FW) respectively. Overall, the predicted trends were found to be in accordance with established knitting theories and mathematical relationships governing fabric geometry, areal density, and dimensional characteristics [37,38].

### Assessment and comparison of the model

**Fig 10:**
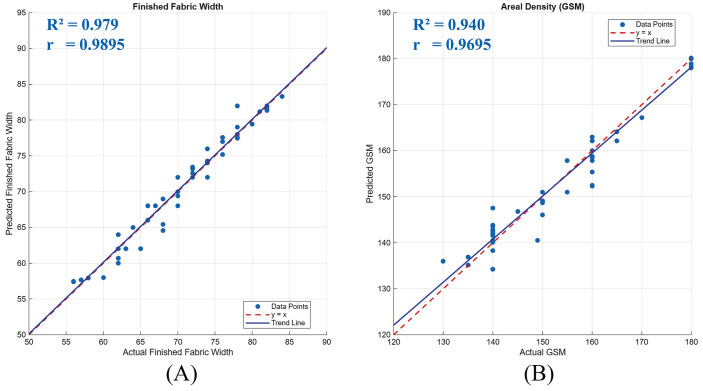
Scatter plot between, (A) Actual FW and Predicted FW, (B) Actual GSM and Predicted GSM.

Fabric samples were knitted using arbitrary input parameters (SL, YC and D) to obtain Finished Fabric Width (FW) and Areal Density (GSM). The model was assessed and compared using the newly developed datasets. The correlation analysis comparing the predicted FW and actual FW with the trend line is shown in **Fig 10(A)**. The MAPE, RMSE and R^2^, of actual data and the predicted data of Finished Fabric Width (FW) were found to be 1.214%, 1.103 and 0.979, using equation 8, 9 and 10 respectively. And to validate the predictive model for Finished Fabric Width (FW), an ANOVA-based F-test was conducted to determine whether any statistically significant difference exists between the predicted and actual values. The results are shown in **Appendix A – Prediction performance of fuzzy model for Finished Fabric Width (FW)** in S1 Appendix of the Supporting Information.

In case of Areal Density (GSM) the correlation analysis is plotted in **Fig 10(B)**. The MAPE, RMSE and R^2^, of actual data and the predicted data of GSM were found to be 1.661%, 3.892 and 0.940 using equation 8, 9 and 10 respectively. And for the validation of the predictive model developed for Areal Density (GSM), an ANOVA-based F-test was employed to investigate whether any statistically significant deviation exists between the predicted and actual values. The results are shown in **Appendix B – Prediction performance of fuzzy model for Areal Density (GSM)** in S1 Appendix of the Supporting Information.

The results presented in Appendix A: Prediction performance of fuzzy model for Finished Fabric Width and Appendix B: Prediction performance of fuzzy model for Areal Density (GSM), show excellent predictive performance of the developed model for Finished Fabric Width (FW) and Areal Density (GSM). The calculated F-values for FW and GSM were 0.016 and 0.000347 respectively. These values are substantially lower than the corresponding critical F-values at conventional significance levels of α = 0.10, α = 0.05, α = 0.01, and α = 0.001. Therefore, confirming no statistically significant differences between the predicted and actual values at confidence levels of 90%, 95%, 99%, and 99.9%. The exceptionally low F-values further highlights the outstanding predictive performance of the developed model for FW and GSM.

**Fig 11:**
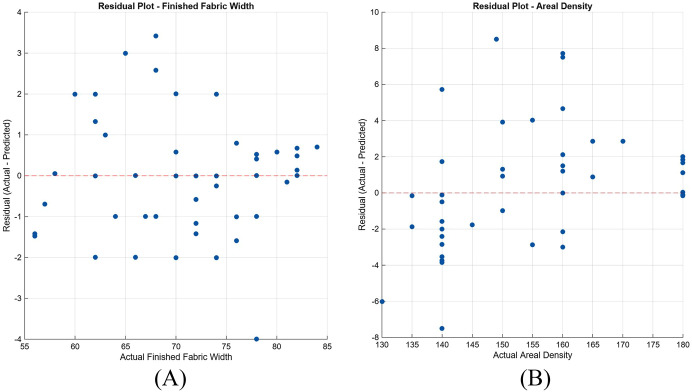
Residual plot for, (A) Finished Fabric Width, (B) Areal Density.

Fig 11 represents the residual plots used to evaluate model capability. **Fig 11(A)**. corresponds to the FW model, while **Fig 11(B)**. depicts the GSM model. In both cases, the residuals are plotted against the actual values, with the horizontal line at zero serving as a reference. The distribution of points is largely scattered around this line without a distinct pattern, suggesting that the assumptions of homoscedasticity and linearity are reasonably satisfied. No major systematic deviations are visible, indicating that the model provide an adequate fit to the data.

In addition to achieving high predictive accuracy, the developed fuzzy model demonstrates several distinctive advantages over prior approaches. First, the model structure is characteristically interpretable, allowing the relationship between knitting parameters (SL, YC, and D) and key fabric properties (FW and GSM) to be expressed in a transparent rule-based format. This interpretability is particularly valuable for textile engineers, as it facilitates direct insights into process-property linkages rather than treating the model as a “black box” system. Second, the model achieves this performance with a compact set of rules, thereby reducing redundancy and enhancing applicability across different datasets. Compared to other data-driven techniques such as neural networks or conventional regression-based models, the proposed fuzzy framework captures nonlinear relationships between knitting parameters and fabric properties with fewer assumptions that requires substantially lower computational resources while maintaining competitive accuracy. This efficiency makes it well- suited for integration into industrial environments, where rapid prediction and decision-making are critical.

Looking ahead, The framework could be extended for blended yarn fabrics (e.g., CVC, PC.), alternative knit structures (e.g., Single Lacoste, Polo Pique, Rib, Interlock.), or dynamic production environments with different machine gauges, where real-time adjustments are needed. Such extensions would further demonstrate the adaptability of the fuzzy approach and broaden its applicability across diverse textile manufacturing context.

## Conclusion

In this study, a fuzzy logic model was developed to predict the finished fabric width and areal density of single jersey circular knitted 100% cotton fabric. The model considered stitch length, yarn count, and machine diameter as input variables. It showed excellent prediction accuracy and aligned well with real-world experimental results. Moreover, the model helped to uncover how each input affects the fabric properties. Both stitch length and yarn count were found to have a negative effect on areal density, while finished fabric width increased significantly with machine diameter. Moreover, Machine Dia and Stitch Length did not contribute to a mentionable change in Areal Density and Finished Fabric Width respectively. To ensure the reliability of the model, it was validated using the most recent experimental data. The performance was measured using Mean Absolute Percentage Error (MAPE), Root Mean Square Error (RMSE), and the Coefficient of Determination (R²). For finished fabric width, the values were 1.214, 1.103, and 0.979, respectively. For areal density, the values were 1.661, 3.892, and 0.940, respectively. These results reflect the model’s strong ability to predict fabric properties accurately.

Overall, the findings suggest that this fuzzy model can be practical and efficient in industrial textiles sector for predicting finished fabric width and areal density of single jersey cotton fabrics produced on circular weft knitting machines. Given the high accuracy achieved by the model and its performance based on industry-scale datasets, It can be inferred that the model has the potential to replace extensive repositories currently used in the textile industries, while also managing production planning and control before initiating the process. Beyond demonstrating strong predictive accuracy, the fuzzy model offers distinct advantages compared to conventional approaches. The rule-based structure provides interpretability, enabling a clearer understanding of how input parameters influence Finished Fabric Width and GSM. Moreover, The model achieves these results with a compact rule set, avoiding unnecessary complexity and improving applicability. Its computational efficiency further enhances suitability for real-time industrial applications. Taken together, these features highlight the novelty of the present approach. It not only matches or exceeds the predictive power of existing models but also provides a transparent, compact, and computationally efficient solution tailored to practical textile manufacturing applications.

## Supporting information

S1 Fig Fuzzy Inference Model System. (TIF)

S2 Fig Model showing results for SL-28.5, YC-22, and D-28. (TIF)

S3 Fig Model showing results for SL- 27, YC – 26, and D-30. (TIF)

S4 Fig Model showing results for SL-27, YC-30, and D-32. (TIF)

S5 Fig Model showing results for SL-28.5, YC-28 and D-34. (TIF)

S6 Fig Model showing results for SL-27, YC-34, and D-36. (TIF)

S7 Fig Model showing results for SL-29, YC- 24, and D-40. (TIF)

S1 File Graphical Abstract. (PDF)

S2 File Generated Fuzzy Rules. (XLSX)

S1 Appendix Appendix A – Prediction performance of fuzzy model for Finished Fabric Width (FW) and Appendix B – Prediction performance of fuzzy model for Areal Density (GSM). (DOCX)
