# Supplementary figures and images for: Predicting the finished fabric width and areal density (Grams per Square Meter) of commercially produced plain Single Jersey (100% Cotton) Knitted Fabric using Fuzzy Inference System (FIS)
Source: PLoS One. 2026 Jul 9;21(7):e0345720. doi: 10.1371/journal.pone.0345720 (PMC13349152; doi:10.1371/journal.pone.0345720)

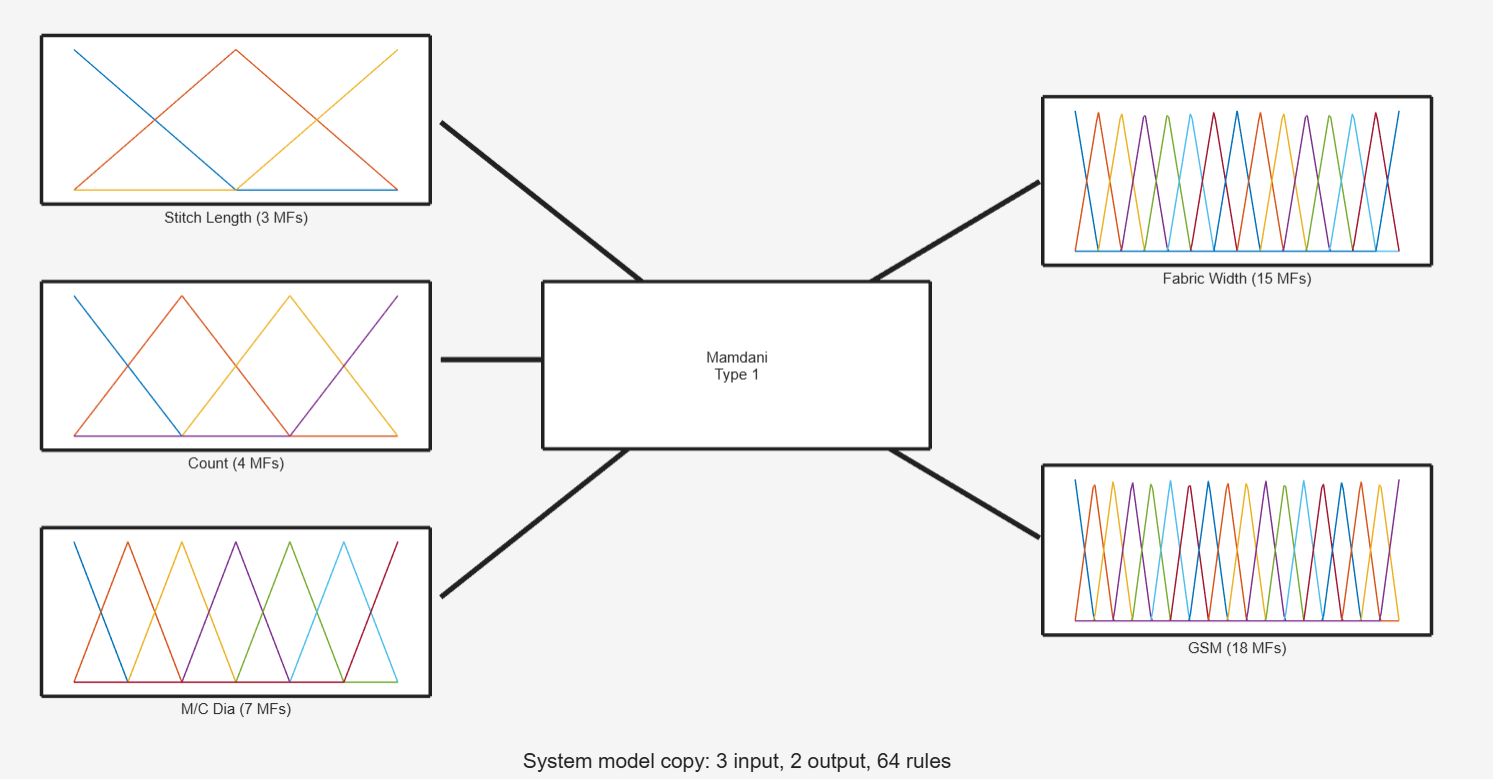

Supplement: S1 Fig — (TIF) [file pone.0345720.s001.tif]

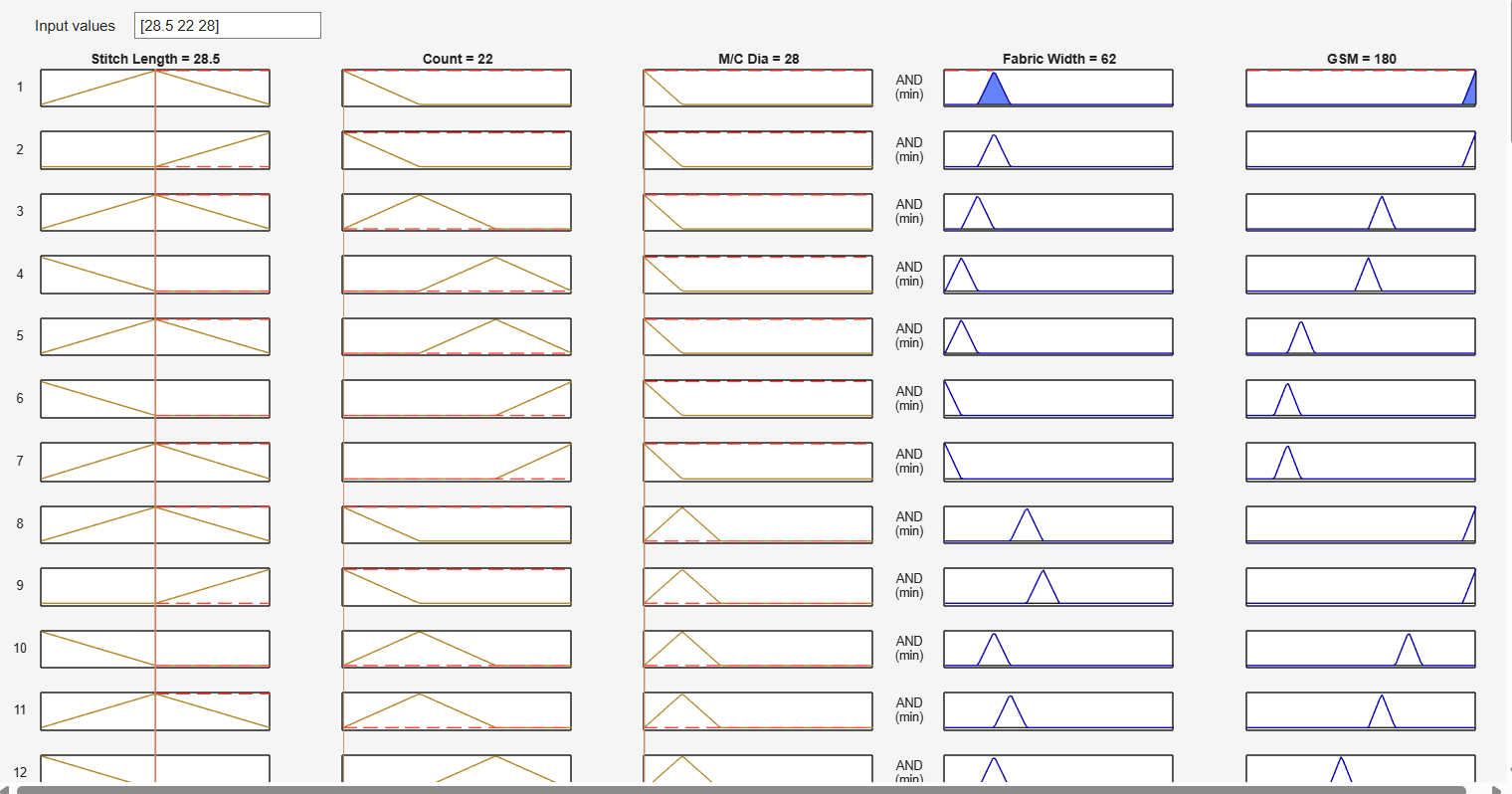

Supplement: S2 Fig — (TIF) [file pone.0345720.s002.tif]

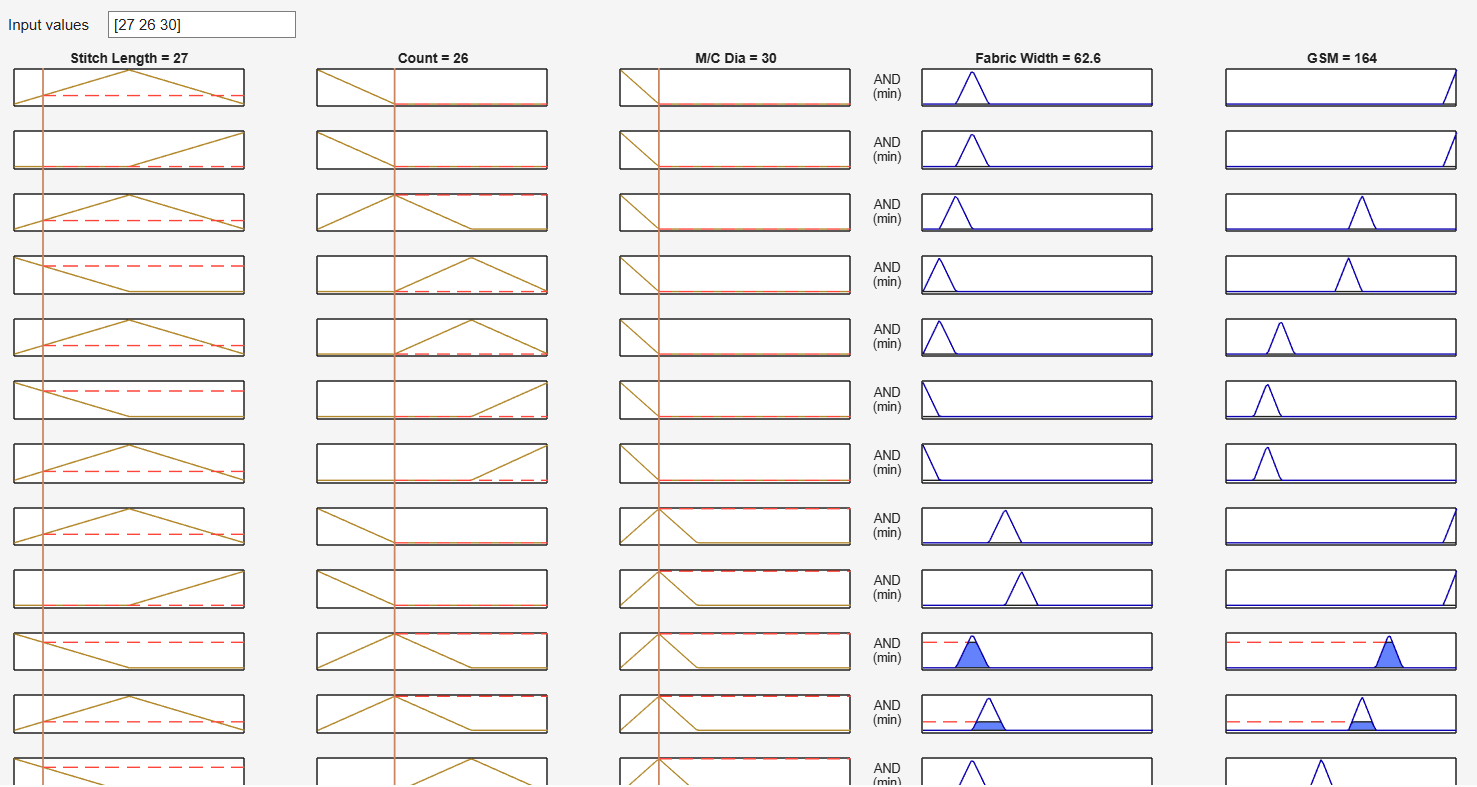

Supplement: S3 Fig — (TIF) [file pone.0345720.s003.tif]

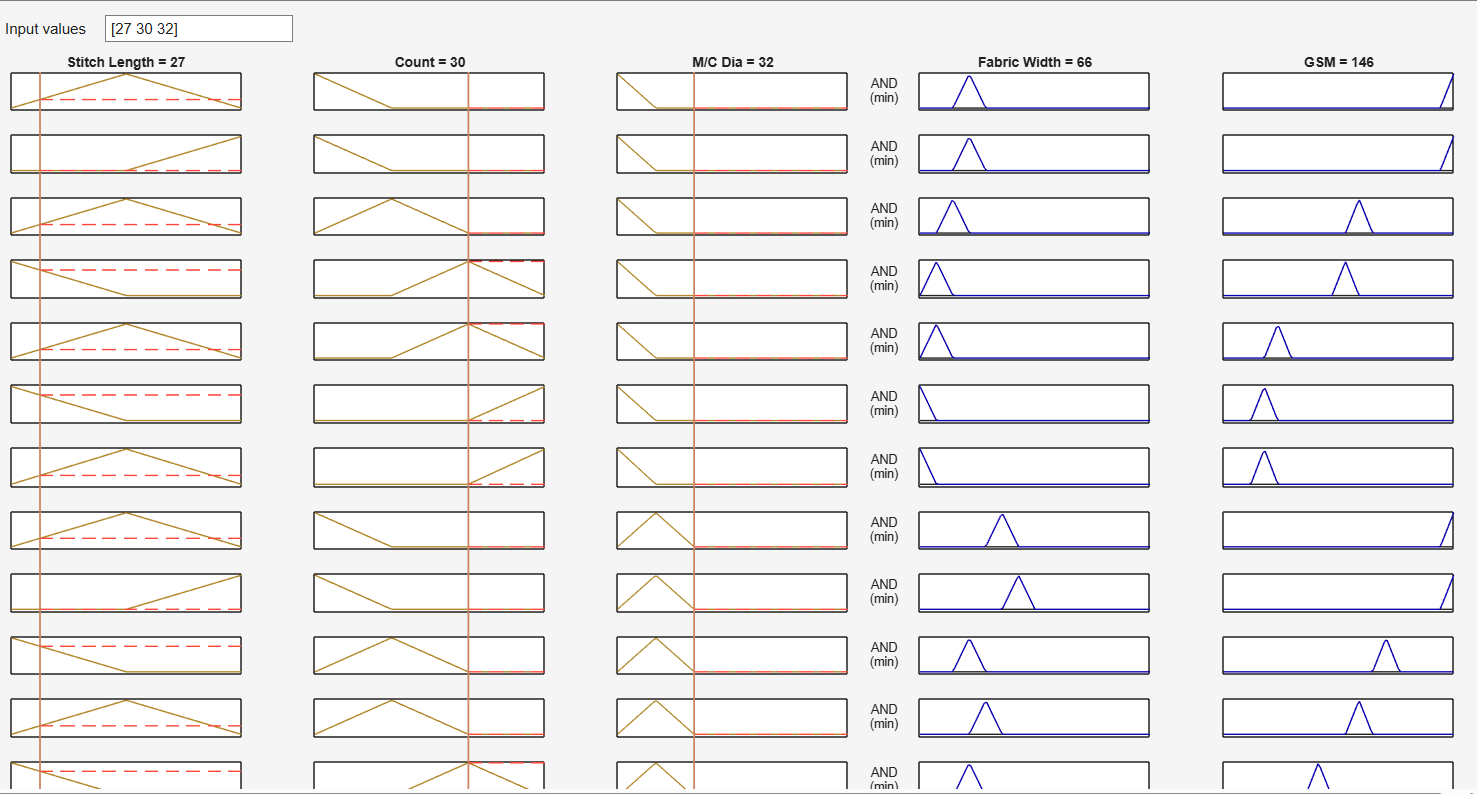

Supplement: S4 Fig — (TIF) [file pone.0345720.s004.tif]

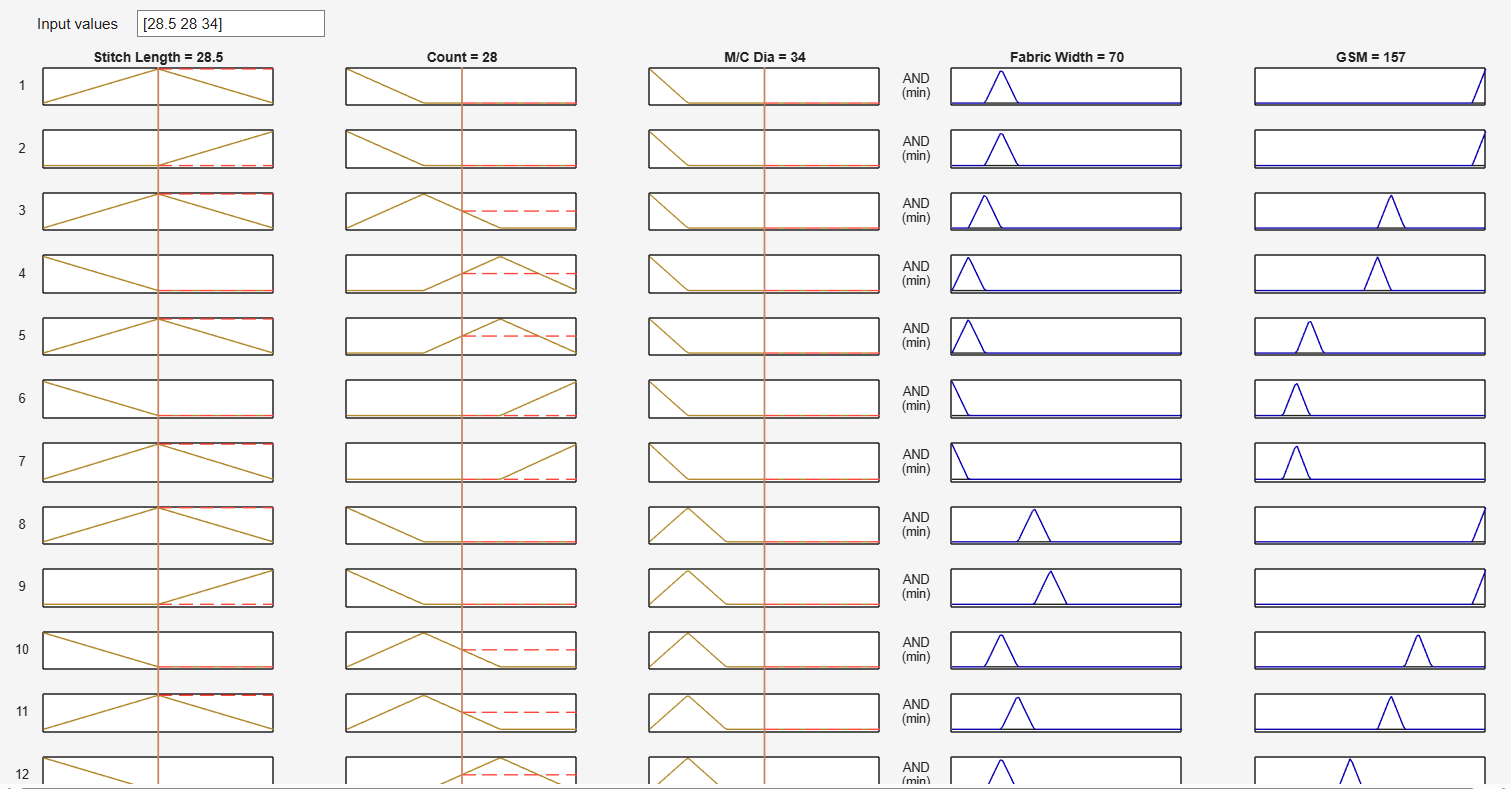

Supplement: S5 Fig — (TIF) [file pone.0345720.s005.tif]

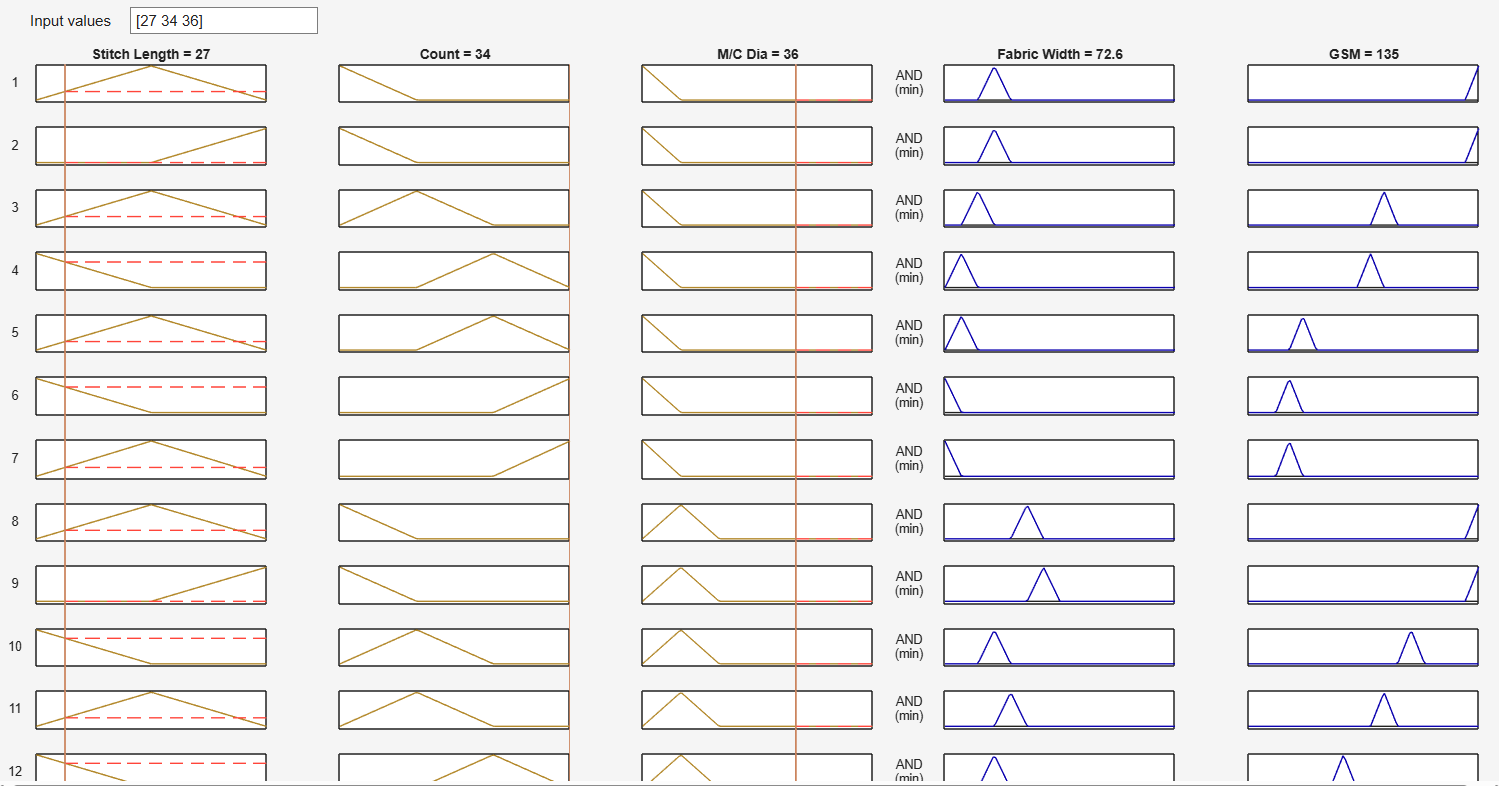

Supplement: S6 Fig — (TIF) [file pone.0345720.s006.tif]

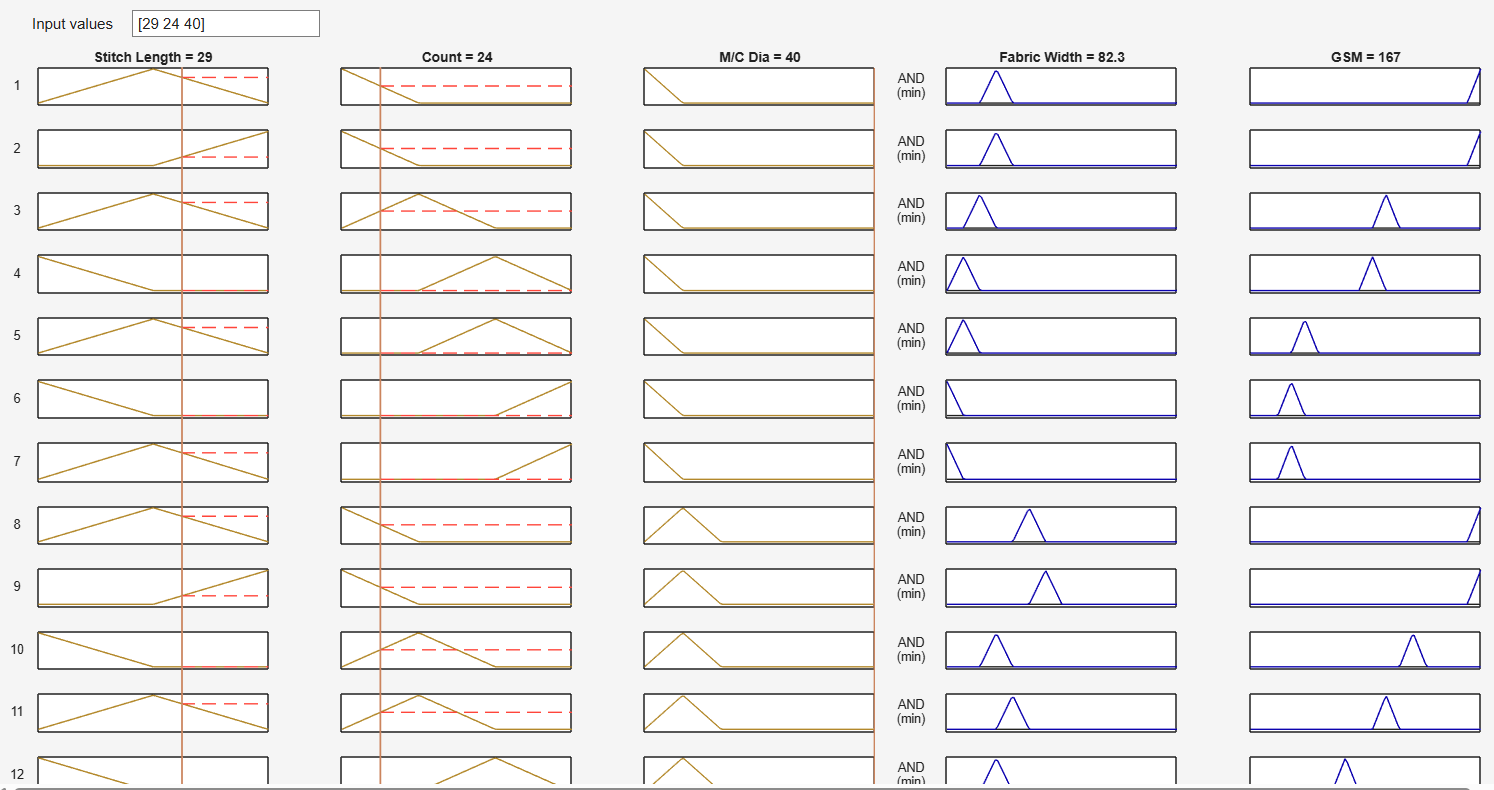

Supplement: S7 Fig — (TIF) [file pone.0345720.s007.tif]
