# Supplementary material for: Predicting the finished fabric width and areal density (Grams per Square Meter) of commercially produced plain Single Jersey (100% Cotton) Knitted Fabric using Fuzzy Inference System (FIS)
Source: PLoS One. 2026 Jul 9;21(7):e0345720. doi: 10.1371/journal.pone.0345720 (PMC13349152; doi:10.1371/journal.pone.0345720)
Supplement: S1 File — (PDF) [file pone.0345720.s008.pdf]

Predicting The Finished Fabric Width & Areal Density (Grams per Square Meter) of Plain Single Jersey Fabric Using FUZZY INFERENCE SYSTEM (FIS)

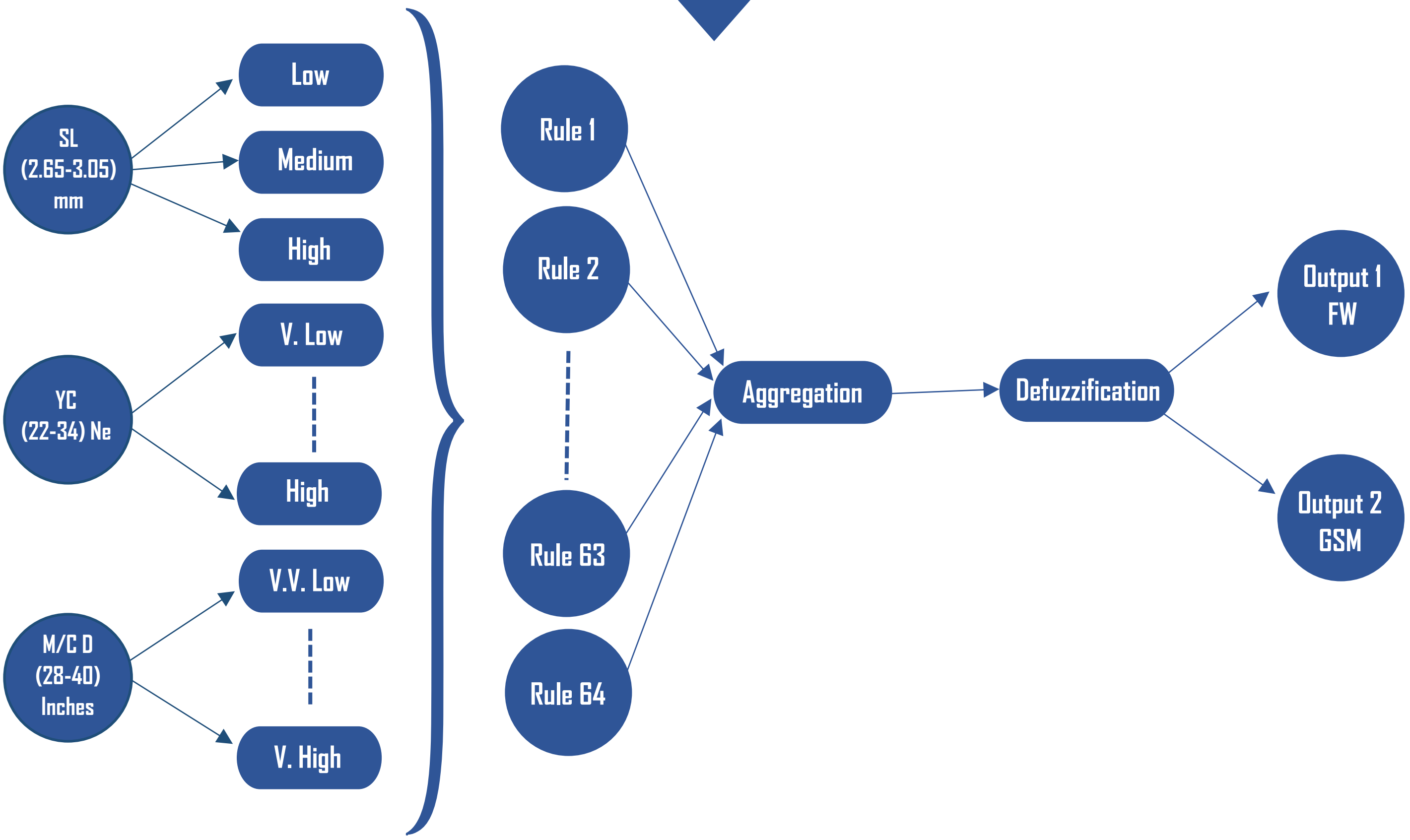

Validation & Comparison of Fuzzy Model

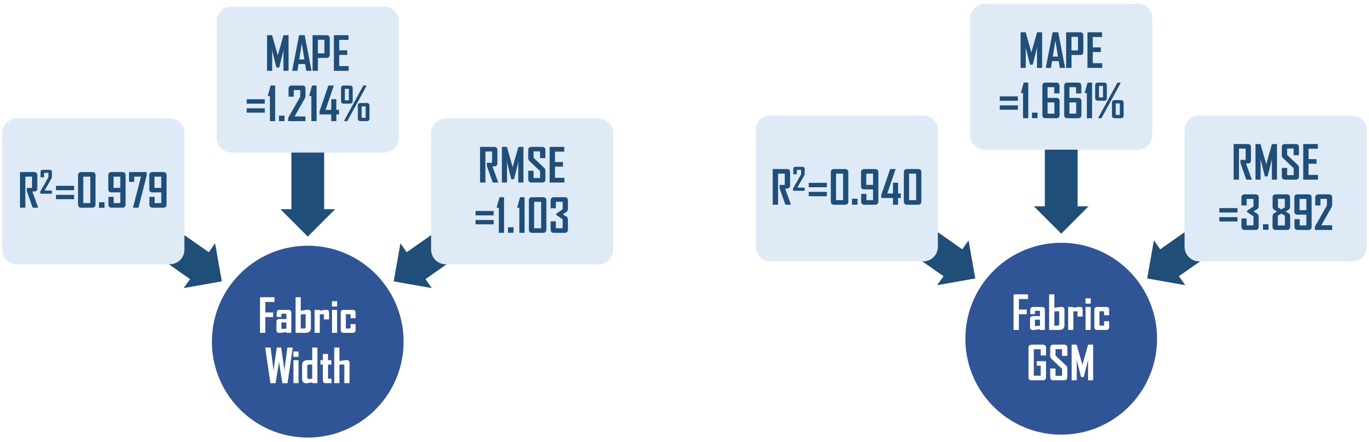

SUMMARY: The FUZZY Model can be a Practical & Effective Tool for Predicting Fabric Width & GSM in Single Jersey Cotton Fabrics
