# Supplementary material for: Predicting the finished fabric width and areal density (Grams per Square Meter) of commercially produced plain Single Jersey (100% Cotton) Knitted Fabric using Fuzzy Inference System (FIS)
Source: PLoS One. 2026 Jul 9;21(7):e0345720. doi: 10.1371/journal.pone.0345720 (PMC13349152; doi:10.1371/journal.pone.0345720)
Supplement: S1 Appendix — (DOCX) [file pone.0345720.s010.docx]

**Appendix A -** Prediction performance of fuzzy model for Finished Fabric Width (FW)

| Sl.  No. | Stitch Length  (mm) | | Yarn  Count  (Ne) | Machine Diameter  (Inch) | | Actual FW (A_d_)  (Inch) | | Predicted  FW (P_d_)  (Inch) | Difference  (A_d_-P_d_) | | Absolute Error  (%) |
| --- | --- | --- | --- | --- | --- | --- | --- | --- | --- | --- | --- |
| 01 | 28.5 | | 22 | 28 | | 62 | | 62 | 0 | | 0.000 |
| 02 | 30.5 | | 22 | 28 | | 62 | | 62 | 0 | | 0.000 |
| 03 | 28 | | 26 | 28 | | 60 | | 60 | 0 | | 0.000 |
| 04 | 27 | | 30 | 28 | | 58 | | 57.9 | 0.1 | | 0.172 |
| 05 | 29 | | 30 | 28 | | 56 | | 57.4 | -1.4 | | 2.500 |
| 06 | 27 | | 32 | 28 | | 57 | | 57.7 | -0.7 | | 1.228 |
| 07 | 28 | | 32 | 28 | | 56 | | 57.5 | -1.5 | | 2.679 |
| 08 | 29 | | 22 | 30 | | 68 | | 66.6 | 1.4 | | 2.059 |
| 09 | 30 | | 22 | 30 | | 68 | | 67.4 | 0.6 | | 0.882 |
| 10 | 27 | | 26 | 30 | | 63 | | 62.6 | 0.4 | | 0.635 |
| 11 | 29 | | 26 | 30 | | 65 | | 64 | 1 | | 1.538 |
| 12 | 26.5 | | 30 | 30 | | 62 | | 62 | 0 | | 0.000 |
| 13 | 28.5 | | 30 | 30 | | 62 | | 60.7 | 1.3 | | 2.097 |
| 14 | 28 | | 34 | 30 | | 62 | | 62 | 0 | | 0.000 |
| 15 | 28 | | 22 | 32 | | 70 | | 72 | -2 | | 2.857 |
| 16 | 30 | | 22 | 32 | | 74 | | 72 | 2 | | 2.703 |
| 17 | 27 | | 26 | 32 | | 67 | | 68 | -1 | | 1.493 |
| 18 | 28.5 | | 26 | 32 | | 66 | | 68 | -2 | | 3.030 |
| 19 | 30 | | 26 | 32 | | 66 | | 68 | -2 | | 3.030 |
| 20 | 27 | | 30 | 32 | | 66 | | 66 | 0 | | 0.000 |
| 21 | 28.5 | | 30 | 32 | | 66 | | 66 | 0 | | 0.000 |
| 22 | 27 | | 34 | 32 | | 62 | | 62.6 | -0.6 | | 0.968 |
| 23 | 28.5 | | 32 | 32 | | 64 | | 65 | -1 | | 1.563 |
| 24 | 28.5 | | 24 | 34 | | 74 | | 73 | 1 | | 1.351 |
| 25 | 30 | | 22 | 34 | | 74 | | 74 | 0 | | 0.000 |
| 26 | 27 | | 26 | 34 | | 70 | | 70.6 | -0.6 | | 0.857 |
| 27 | 29 | | 26 | 34 | | 70 | | 72 | -2 | | 2.857 |
| 28 | 30 | | 26 | 34 | | 72 | | 72 | 0 | | 0.000 |
| 29 | 27 | | 30 | 34 | | 70 | | 69.4 | 0.6 | | 0.857 |
| 30 | 28.5 | | 28 | 34 | | 70 | | 70 | 0 | | 0.000 |
| 31 | 27 | | 32 | 34 | | 68 | | 69 | -1 | | 1.471 |
| 32 | 28.5 | | 32 | 34 | | 70 | | 68 | 2 | | 2.857 |
| 33 | 29 | | 22 | 36 | | 78 | | 77.5 | 0.5 | | 0.641 |
| 34 | 29.5 | | 22 | 36 | | 78 | | 77.6 | 0.4 | | 0.513 |
| 35 | 27.5 | | 28 | 36 | | 74 | | 74.3 | -0.3 | | 0.405 |
| 36 | 28.5 | | 26 | 36 | | 74 | | 76 | -2 | | 2.703 |
| 37 | 29.5 | | 26 | 36 | | 76 | | 75.2 | 0.8 | | 1.053 |
| 38 | 27.5 | | 30 | 36 | | 72 | | 73.4 | -1.4 | | 1.944 |
| 39 | 28.5 | | 30 | 36 | | 72 | | 73.2 | -1.2 | | 1.667 |
| 40 | 27 | | 34 | 36 | | 72 | | 72.6 | -0.6 | | 0.833 |
| 41 | 28.5 | | 32 | 36 | | 72 | | 73.4 | -1.4 | | 1.944 |
| 42 | 28 | | 22 | 38 | | 78 | | 80 | -2 | | 2.564 |
| 43 | 29.5 | | 22 | 38 | | 82 | | 80 | 2 | | 2.439 |
| 44 | 27.5 | | 26 | 38 | | 78 | | 79 | -1 | | 1.282 |
| 45 | 28 | | 26 | 38 | | 80 | | 79.4 | 0.6 | | 0.750 |
| 46 | 27 | | 30 | 38 | | 78 | | 78 | 0 | | 0.000 |
| 47 | 28.5 | | 30 | 38 | | 78 | | 78 | 0 | | 0.000 |
| 48 | 27 | | 34 | 38 | | 76 | | 77 | -1 | | 1.316 |
| 49 | 28 | | 32 | 38 | | 76 | | 77.6 | -1.6 | | 2.105 |
| 50 | 29 | | 24 | 40 | | 84 | | 82.3 | 1.7 | | 2.024 |
| 51 | 27.5 | | 28 | 40 | | 82 | | 81.5 | 0.5 | | 0.610 |
| 52 | 28.5 | | 28 | 40 | | 82 | | 81.5 | 0.5 | | 0.610 |
| 53 | 27.5 | | 30 | 40 | | 82 | | 81.5 | 0.5 | | 0.610 |
| 54 | 28.5 | | 30 | 40 | | 82 | | 81.3 | 0.7 | | 0.854 |
| 55 | 29 | | 32 | 40 | | 81 | | 81.2 | -0.2 | | 0.247 |
| Mean Absolute Percentage Error (MAPE) | | | | | | | | | 1.214% | | |
| Root Mean Square Error (RMSE) | | | | | | | | | 1.103 | | |
| Coefficient of Determination (R^2^) | | | | | | | | | 0.979 | | |
| ANOVA Summary: | | | | | | | | | | | |
| Source of Variation | | DF | | | Sum of Squares | | Mean Squares | | | Computed F | |
| Between Groups | | 1 | | | 0.891 | | 0.891 | | | 0.016 | |
| Within Groups | | 108 | | | 6023.8262 | | 55.7762 | | |  |  |
| Total | | 109 | | | 6024.7172 | |  | | |  |  |

**Appendix B -** Prediction performance of fuzzy model for Areal Density (GSM)

| Sl.  No. | Stitch Length  (mm) | Yarn  Count  (Ne) | Machine Diameter  (Inch) | Actual Areal Density  (GSM) (A_d_) | Predicted  Areal Density  (GSM) (P_d_) | Difference  (A_d_ – P_d_) | Absolute  Error  (%) |
| --- | --- | --- | --- | --- | --- | --- | --- |
| 01 | 28.5 | 22 | 28 | 180 | 180 | 0 | 0.000 |
| 02 | 30.5 | 22 | 28 | 180 | 180 | 0 | 0.000 |
| 03 | 28 | 26 | 28 | 160 | 160 | 0 | 0.000 |
| 04 | 27 | 30 | 28 | 150 | 153 | -3 | 2.000 |
| 05 | 29 | 30 | 28 | 140 | 142 | -2 | 1.429 |
| 06 | 27 | 32 | 28 | 140 | 148 | -8 | 5.714 |
| 07 | 28 | 32 | 28 | 140 | 145 | -5 | 3.571 |
| 08 | 29 | 22 | 30 | 180 | 180 | 0 | 0.000 |
| 09 | 30 | 22 | 30 | 180 | 180 | 0 | 0.000 |
| 10 | 27 | 26 | 30 | 165 | 164 | 1 | 0.606 |
| 11 | 29 | 26 | 30 | 160 | 160 | 0 | 0.000 |
| 12 | 26.5 | 30 | 30 | 150 | 151 | -1 | 0.667 |
| 13 | 28.5 | 30 | 30 | 140 | 139 | 1 | 0.714 |
| 14 | 28 | 34 | 30 | 130 | 139 | -9 | 6.923 |
| 15 | 28 | 22 | 32 | 180 | 180 | 0 | 0.000 |
| 16 | 30 | 22 | 32 | 180 | 180 | 0 | 0.000 |
| 17 | 27 | 26 | 32 | 165 | 164 | 1 | 0.606 |
| 18 | 28.5 | 26 | 32 | 160 | 160 | 0 | 0.000 |
| 19 | 30 | 26 | 32 | 155 | 156 | -1 | 0.645 |
| 20 | 27 | 30 | 32 | 150 | 146 | 4 | 2.667 |
| 21 | 28.5 | 30 | 32 | 140 | 142 | -2 | 1.429 |
| 22 | 27 | 34 | 32 | 135 | 136 | -1 | 0.741 |
| 23 | 28.5 | 32 | 32 | 149 | 141 | 8 | 5.369 |
| 24 | 28.5 | 24 | 34 | 180 | 173 | 7 | 3.889 |
| 25 | 30 | 22 | 34 | 180 | 180 | 0 | 0.000 |
| 26 | 27 | 26 | 34 | 160 | 167 | -7 | 4.375 |
| 27 | 29 | 26 | 34 | 160 | 166 | -6 | 3.750 |
| 28 | 30 | 26 | 34 | 160 | 163 | -3 | 1.875 |
| 29 | 27 | 30 | 34 | 150 | 147 | 3 | 2.000 |
| 30 | 28.5 | 28 | 34 | 160 | 157 | 3 | 1.875 |
| 31 | 27 | 32 | 34 | 140 | 144 | -4 | 2.857 |
| 32 | 28.5 | 32 | 34 | 140 | 142 | -2 | 1.429 |
| 33 | 29 | 22 | 36 | 180 | 179 | 1 | 0.556 |
| 34 | 29.5 | 22 | 36 | 180 | 178 | 2 | 1.111 |
| 35 | 27.5 | 28 | 36 | 160 | 153 | 7 | 4.375 |
| 36 | 28.5 | 26 | 36 | 160 | 160 | 0 | 0.000 |
| 37 | 29.5 | 26 | 36 | 160 | 160 | 0 | 0.000 |
| 38 | 27.5 | 30 | 36 | 140 | 147 | -7 | 5.000 |
| 39 | 28.5 | 30 | 36 | 140 | 142 | -2 | 1.429 |
| 40 | 27 | 34 | 36 | 135 | 135 | 0 | 0.000 |
| 41 | 28.5 | 32 | 36 | 140 | 140 | 0 | 0.000 |
| 42 | 28 | 22 | 38 | 180 | 180 | 0 | 0.000 |
| 43 | 29.5 | 22 | 38 | 180 | 180 | 0 | 0.000 |
| 44 | 27.5 | 26 | 38 | 160 | 159 | 1 | 0.625 |
| 45 | 28 | 26 | 38 | 160 | 159 | 1 | 0.625 |
| 46 | 27 | 30 | 38 | 150 | 148 | 2 | 1.333 |
| 47 | 28.5 | 30 | 38 | 140 | 142 | -2 | 1.429 |
| 48 | 27 | 34 | 38 | 140 | 141 | -1 | 0.714 |
| 49 | 28 | 32 | 38 | 140 | 142 | -2 | 1.429 |
| 50 | 29 | 24 | 40 | 180 | 167 | 13 | 7.222 |
| 51 | 27.5 | 28 | 40 | 160 | 153 | 7 | 4.375 |
| 52 | 28.5 | 28 | 40 | 155 | 149 | 6 | 3.871 |
| 53 | 27.5 | 30 | 40 | 145 | 144 | 1 | 0.690 |
| 54 | 28.5 | 30 | 40 | 140 | 139 | 1 | 0.714 |
| 55 | 29 | 32 | 40 | 140 | 139 | 1 | 0.714 |
| Mean Absolute Percentage Error (MAPE) | | | | | | 1.661% | |
| Root Mean Square Error (RMSE) | | | | | | 3.892 | |
| Coefficient of Determination (R^2^) | | | | | | 0.940 | |

| ANOVA Summary: | | | | |
| --- | --- | --- | --- | --- |
| Source of Variation | DF | Sum of Squares | Mean Squares | Computed F |
| Between Groups | 1 | 0.0818 | 0.0818 | 0.000347 |
| Within Groups | 108 | 25472.5091 | 235.8566 |  |
| Total | 109 | 25472.5909 |  |  |
